# Editors at the frontier: from the editorial desk to the research lab

**DOI:** 10.1038/s41377-026-02191-y

**Published:** 2026-02-06

**Authors:** Siqiu Guo, Fei Ding

**Affiliations:** 1 Light Publishing Group, Changchun Institute of Optics, Fine Mechanics and Physics, Chinese Academy of Sciences, 3888 Dong Nan Hu Road, Changchun, 130033 China; 2 Ji Hua Laboratory, 28 Huandao South Road, Foshan, 528200 China; 3 Leibniz University Hannover, Appelstr. 2, Hannover, 30559 Germany

## Abstract

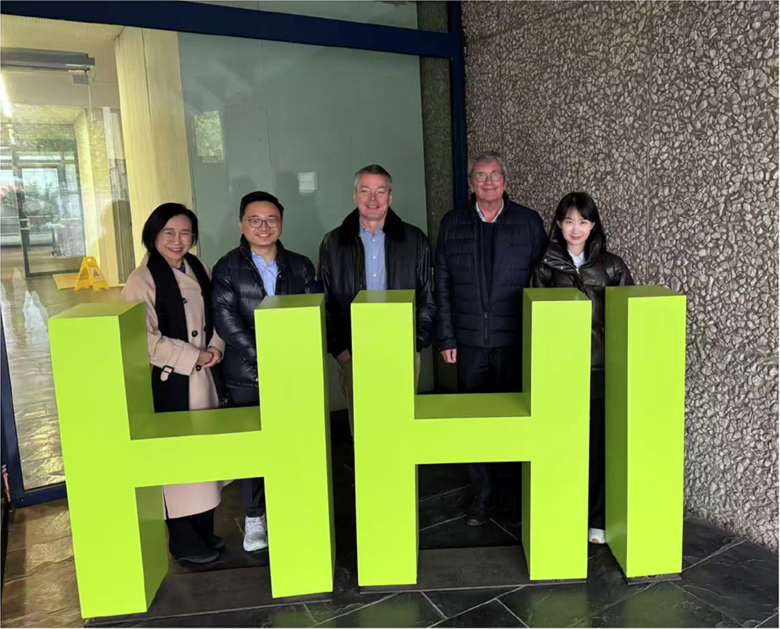

On 20 November 2025, at the invitation of Prof. Dieter Bimberg—a member of the German National Academy of Sciences Leopoldina, the US National Academy of Engineering, and the Russian Academy of Sciences, and a Fellow of the National Academy of Inventors (USA)—a delegation from *Light: Science & Applications* (*Light*) paid a visit to the Fraunhofer Heinrich Hertz Institute (HHI) in Berlin. The delegation comprised Prof. Yuhong Bai, Executive Editor-in-Chief and founder of the Light brand; Prof. Fei Ding (Leibniz University Hannover), Head of the Light Hannover Office; and Prof. Siqiu Guo, Deputy Executive Editor-in-Chief of the Light Publishing Group (Fig. 1).

**Fig. 1:**
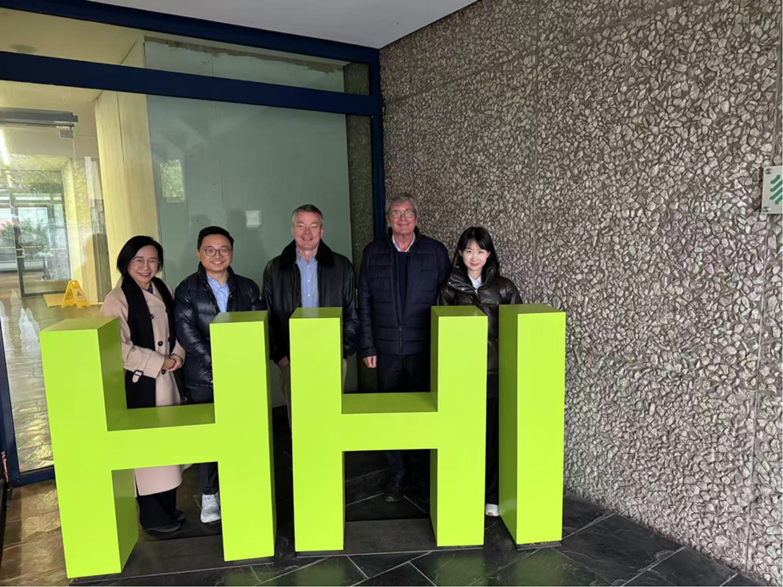
The *Light* delegation visiting HHI (from left to right: Prof. Yuhong Bai, Prof. Fei Ding, Prof. Martin Schell, Prof. Dieter Bimberg, and Prof. Siqiu Guo)

During the visit, Prof. Martin Schell, Executive Director of HHI, introduced the institute’s long-term development vision and its current research priorities, spanning integrated photonics, high-speed optical communications, photonic integration platforms, and next-generation optoelectronic systems. These research directions, deeply embedded in Europe’s photonics ecosystem, resonate strongly with global efforts to push the limits of light-based technologies in information, sensing, and manufacturing.

This visit was not a ceremonial exchange, nor merely an institutional courtesy. It reflects a conscious repositioning of the role of scientific editors—particularly those representing China’s rapidly evolving science and technology journals. In an era when scientific progress accelerates across disciplines and continents, journals can no longer function solely as passive recipients and evaluators of manuscripts. Instead, they must engage actively with the scientific frontier, listening closely to the questions leading researchers are asking—often well before those questions crystallize into papers.

For *Light*, engaging directly with laboratories and research leaders such as those at HHI serves multiple purposes. First, it enables editors to maintain a first-hand understanding of emerging directions, technological bottlenecks, and conceptual shifts in photonics. Second, it fosters mutual trust and intellectual resonance between journals and the research community—an essential ingredient for attracting transformative work. Third, such dialogue opens pathways for new forms of collaboration, including thematic issues, perspective articles, and cross-border academic initiatives that can amplify innovative ideas at an early stage.

More broadly, this visit represents an exploratory step for Chinese science journals to engage globally in a substantive way. Internationalization is not achieved merely by publishing in English or assembling an international editorial board. It requires editors to participate in the global circulation of ideas, to understand diverse research cultures, and to engage in sustained, two-way conversations with scientists at the forefront of discovery. By doing so, journals become not only platforms for dissemination but also interfaces where scientific trajectories are shaped.

The discussions in Berlin reinforced a shared understanding: many of today’s most impactful breakthroughs in photonics arise at the intersection of materials, device physics, integration technologies, and system-level thinking. Capturing such interdisciplinarity demands editorial sensitivity, strategic vision, and an openness to new formats and narratives. For *Light*, this reinforces our commitment to continuously refine our editorial scope, standards, and modes of engagement—ensuring that the journal remains aligned with the evolving landscape of optical science and applications.

As *Light* moves forward, visits such as this will continue to inform how we identify emerging topics, cultivate international partnerships, and support the global photonics community. By staying close to the scientific frontier and maintaining dialogue with its leaders, we aim not only to reflect progress but to help illuminate the paths ahead.

